# Polθ Inhibitor (ART558) Demonstrates a Synthetic Lethal Effect with PARP and RAD52 Inhibitors in Glioblastoma Cells

**DOI:** 10.3390/ijms25179134

**Published:** 2024-08-23

**Authors:** Gabriela Barszczewska-Pietraszek, Piotr Czarny, Małgorzata Drzewiecka, Maciej Błaszczyk, Maciej Radek, Ewelina Synowiec, Paulina Wigner-Jeziorska, Przemysław Sitarek, Janusz Szemraj, Tomasz Skorski, Tomasz Śliwiński

**Affiliations:** 1 Department of Molecular Genetics, Faculty of Biology and Environmental Protection, University of Lodz, 90-236 Lodz, Poland; gabriela.barszczewska.pietraszek@edu.uni.lodz.pl (G.B.-P.); 2 Department of Medical Biochemistry, Medical University of Lodz, 92-216 Lodz, Poland; 3 Department of Neurosurgery, Surgery of Spine and Peripheral Nerves, Medical University of Lodz, University Hospital WAM-CSW, 90-549 Lodz, Poland; 4 Department of Medical Biology, Medical University of Lodz, 92-151 Lodz, Poland; 5 Fels Cancer Institute for Personalized Medicine, Lewis Katz School of Medicine, Temple University, Philadelphia, PA 19140, USA

**Keywords:** Polθ, RAD52, PARP1 inhibitors, synthetic lethality, DNA repair, DNA damage, glioblastoma, personalized therapy

## Abstract

DNA repair proteins became the popular targets in research on cancer treatment. In our studies we hypothesized that inhibition of DNA polymerase theta (Polθ) and its combination with Poly (ADP-ribose) polymerase 1 (PARP1) or RAD52 inhibition and the alkylating drug temozolomide (TMZ) has an anticancer effect on glioblastoma cells (GBM21), whereas it has a low impact on normal human astrocytes (NHA). The effect of the compounds was assessed by analysis of cell viability, apoptosis, proliferation, DNA damage and cell cycle distribution, as well as gene expression. The main results show that Polθ inhibition causes a significant decrease in glioblastoma cell viability. It induces apoptosis, which is accompanied by a reduction in cell proliferation and DNA damage. Moreover, the effect was stronger when dual inhibition of Polθ with PARP1 or RAD52 was applied, and it is further enhanced by addition of TMZ. The impact on normal cells is much lower, especially when considering cell viability and DNA damage. In conclusion, we would like to highlight that Polθ inhibition used in combination with PARP1 or RAD52 inhibition has great potential to kill glioblastoma cells, and shows a synthetic lethal effect, while sparing normal astrocytes.

## 1. Introduction

Glioblastoma (GBM) previously known as Glioblastoma Multiforme is now recognized as Glioblastoma *Isocitrate dehydrogenase (IDH)* wild-type, according to the WHO classification of Tumors of the Central Nervous System from 2021, and is treated separately from Astrocytoma, *IDH*-mutant tumors, which is different than in previous WHO classifications [1,2]. GBM is an adult-type, diffuse, grade IV glioma and is considered the most common and dangerous brain cancer. The median survival time for a diagnosed patient is 15 months [1,3,4,5]. Primary glioblastoma is formed from glial cells and can be characterized by one of these changes: microvascular proliferation, necrosis, *EGFR* amplification, *TERT* promoter mutation, or combined gain of chromosome 7/loss of chromosome 10 copy number [1,2].

Traditional treatment of GBM includes surgical resection, if possible, radiotherapy and chemotherapy, in which the first recommendation is therapy with temozolomide (TMZ), a commercially available drug approved by the Food and Drug Administration (FDA) and the European Medical Agency (EMA) [3,4,6]. TMZ was found to promote methylation of DNA—the creation of O6-methylguanine—which leads to DNA damage and apoptosis in tumor cells [7,8,9]. We used TMZ in our research due to its widespread use in patients’ treatments. Our objective was to assess its effectiveness with simultaneous inhibition of DNA repair proteins, in the context of a potential combined therapy and its impact on healthy tissues. 

Despite existing possibilities, there are many barriers in the treatment of GBM, such as the localization of the tumor, its spreading characteristics, hindered drug delivery, heterogeneity of the tumor tissue and the continuous issue of tumor recurrence and drug resistance [2]. Therefore, the need for the development of new more precise and efficient treatments is urgent.

The approach of personalized therapy is currently developing in oncology. Studies analyzing molecular markers, gene expression, whole genome sequencing, and epigenetics enable the identification of tumors with particular genetic modifications [10,11]. Mutations in DNA repair pathways, especially of double-strand breaks (DSBs), are important for cancer growth. Cancer cells with repair alterations develop selective growth dominance, combined with genetic instability and further progression. However, usually this makes cancer dependent on one DNA repair pathway, which creates opportunities to defeat tumor cells [11]. The strategy of synthetic lethality (SL) is applied in such cases. The SL is based on affecting two genes simultaneously to kill cells, when silencing either of these genes separately is not lethal [12]. The examples of such a treatment are the small molecule inhibitors targeting proteins involved in DNA DSB repair mechanisms, such as Polθ, PARP1 and Rad52. The inhibitors can be applied in combination to eliminate activity of two or more repair pathways simultaneously, or in tumors with specific deficiencies, e.g., the common homologous recombination (HR) defect or the *BRCA1/2* mutation. The second approach is particularly important for precision medicine since it allows the sparing of nonmalignant cells [13,14,15]. 

The first to obtain significant success in treatment, based on SL interactions, were PARP inhibitors targeting BRCA-mutated cancer cells. Poly (ADP-ribose) polymerases (PARPs) are DNA repair proteins, which play an important role in various processes including HR, base excision repair (BER), classical and alternative non-homologous end joining (NHEJ), nucleotide excision repair (NER), maintenance of replication fork stability, and mismatch repair (MMR). The main representatives are PARP1 and PARP2 proteins [16,17]. There are five different inhibitors that have been approved in the treatment of ovarian and breast cancer in Europe and the United States, i.e., olaparib, rucaparib, niraparib, veliparib and talazoparib [18,19]. Talazoparib was the one used in our study. It is shown that it exhibits a cytotoxic effect on cancer cells via two mechanisms: inhibition of PARP catalytic activity and PARP blockade at the site of DNA damage, thereby stopping further DNA repair and leading to apoptosis and/or cell death [20,21].

Since the success of PARP inhibitors against BRCA-mutated cancer cells, interest in identifying potential SL targets, e.g., Polθ, has grown. However, the tumor’s resistance to PARPi has created an as yet unmet need to expand research in this area [22].

Polθ is a DNA polymerase involved in theta-mediated end joining (TMEJ)—a DSB repair pathway distinct from other mechanisms due to its independence of Ku, XRCC4 and LIG4 proteins, presence of resected DNA ends with 3′ single-stranded overhangs and several nucleotide-long microhomology regions. This is the main activity of Polθ, but not the only one, the protein is engaged in other molecular mechanisms, such as translesion synthesis, base excision repair, mismatch repair, replication-associated DNA breaks, or reverse transcription and interstrand crosslink repair [23,24]. Several Polθ inhibitors, that have gained special interest have been developing in laboratories and clinical studies, i.e., novobiocin, ART558, ART812, ART4215 and RP-6685, described very precisely by Pismataro et al., 2023, with four registered clinical studies (NCT05687110, NCT06077877, NCT04991480, and NCT05898399; clinicaltrials.gov, accessed on 16 January 2024) [11,25,26,27]. ART558 was applied in this study. This inhibitor allosterically anchors to the binding site of Polθ polymerase’s catalytic domain and stabilizes it thermally in the presence of DNA, which together leads to disrupted activity of Polθ [11,25].

The third protein engaged in our study—RAD52 due to its ability to anneal ssDNA—plays a particularly important role in various mechanisms, i.e., HR repair and SSA, replication fork stabilization and assembly of a displacement loop (D-loop). A well-known inhibitor of RAD52, 6-OH-DOPA disassociates the RAD52 ring superstructure converting it to dimers, which results in a repressed protein function [28,29].

The previous studies of our group demonstrated synthetically lethal interaction between a PARP inhibitor (BMN) and class I histone deacetylases, as well as PARPi and LIG4 deficiency in glioblastoma cells [30,31]. To our knowledge this is the first published study demonstrating the influence of Polθi–ART558 and PARPi–BMN673 or RAD52i–L-OH-DOPA on glioblastoma cells.

## 2. Results

### 2.1. Polθ Inhibitor ART558 Used Alone or in Combination with PARP1/RAD52 Inhibitors and the Alkylating Agent TMZ, Lead to Cell Death via Apoptosis in Patient-Derived Glioblastoma Cells

#### 2.1.1. Cell Viability

**Figure 1:**
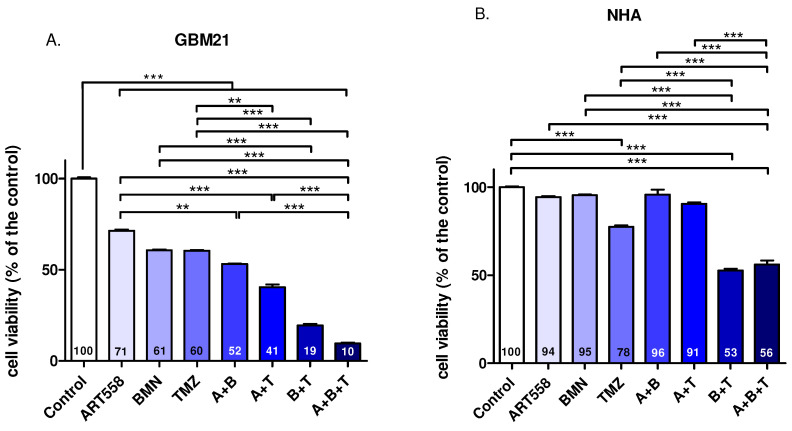

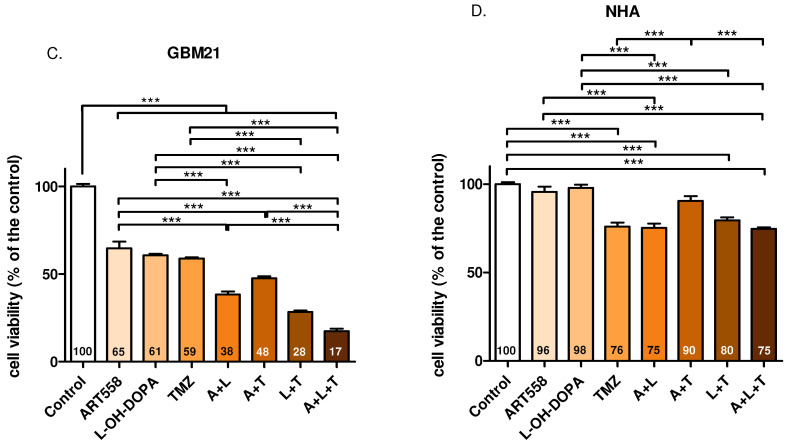
The effect of DNA repair protein inhibition and treatment with an alkylating agent and their combinations on the GBM21 cancer cell line. (**A**)—variant with PARPi, (**C**)—variant with RAD52i and normal NHA (**B**) variant with PARPi, (**D**)—variant with RAD52i cell viability shown as the percentage of the control. The numbers on the bars show the mean values. Three independent experiments were performed and the results are shown as the mean ± standard error of the mean (SEM). ** *p*-value ≤ 0.01, *** *p*-value ≤ 0.001; A—ART558, B—BMN673, L—L-OH-DOPA, T—TMZ.

Inhibition of Polθ or PARP1 or RAD52 produces a significant reduction in glioblastoma cell viability in comparison to the untreated control (Figure 1A,C). The combination of Polθ inhibition with either PARP1 or RAD52 enhances the effect significantly in comparison to the control and to the inhibition of Polθ alone in GBM21. The addition of TMZ to Polθ inhibition produces a significant reduction in GBM21 viability. The strongest effect, and significantly higher than single inhibitors or treatment with TMZ and double combinations, was obtained when three compounds together, either Polθi, PAPRi and TMZ or Polθi, RAD52i and TMZ were applied in both cell lines. However, this effect in GBM21 is around four times stronger than in NHA. Interestingly, considering the single treatment, only TMZ produces a significant reduction in NHA viability, which shows its negative influence on healthy cells. Whereas its combination with Polθi did not cause a significant reduction in NHA viability. Although, double treatment with Polθi and RAD52i decreases NHA cell viability significantly versus the control and Polθi, the reduction in viability is even more pronounced in cancer cells.

#### 2.1.2. Visualization of Morphological Changes by Double Calcein AM/PI Staining

**Figure 2:**
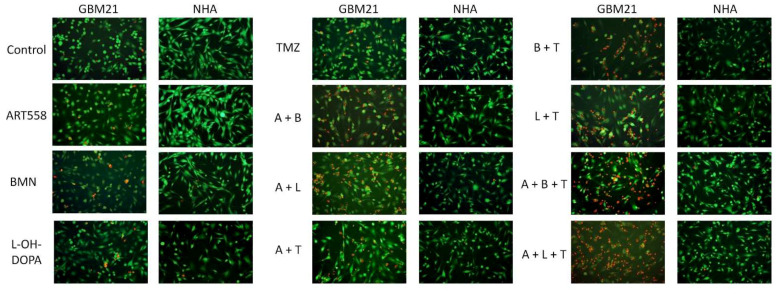
The effect of DNA repair protein inhibition and treatment with an alkylating agent and their combinations on the GBM21 cancer cell line and normal NHA cells, visualized with calcein AM and PI staining. Magnification of the pictures is 10×. A—ART558, B—BMN673, L—L-OH-DOPA, T—TMZ.

Morphological changes induced by Polθ+/− PARP1i/RAD52i/TMZ were assessed by calcein AM/PI double staining (Figure 2). Cells treated with the inhibitors showed the characteristic hallmarks of cellular homeostasis disorders, such as disrupted cellular membrane integrity visible by penetration of PI. Also, a change in the cell size and the general appearance of the cells could be observed. These morphology changes were much more noticeable in cancer than in normal cells, where almost no differences were observed. These alterations in cellular morphology were in agreement with the increasing number of dead cells stained with PI, especially in samples treated with Polθi, TMZ and PAPRi or RAD52i. Based on visual assessment, the evident differences between treatment groups were observed: separate usage of compounds gave around 10% dead cells, dual inhibition and Polθ with TMZ were up to 50%, and the rest of the treatment variants gave more than 50%.

#### 2.1.3. Cell Death Mechanism

**Figure 3:**
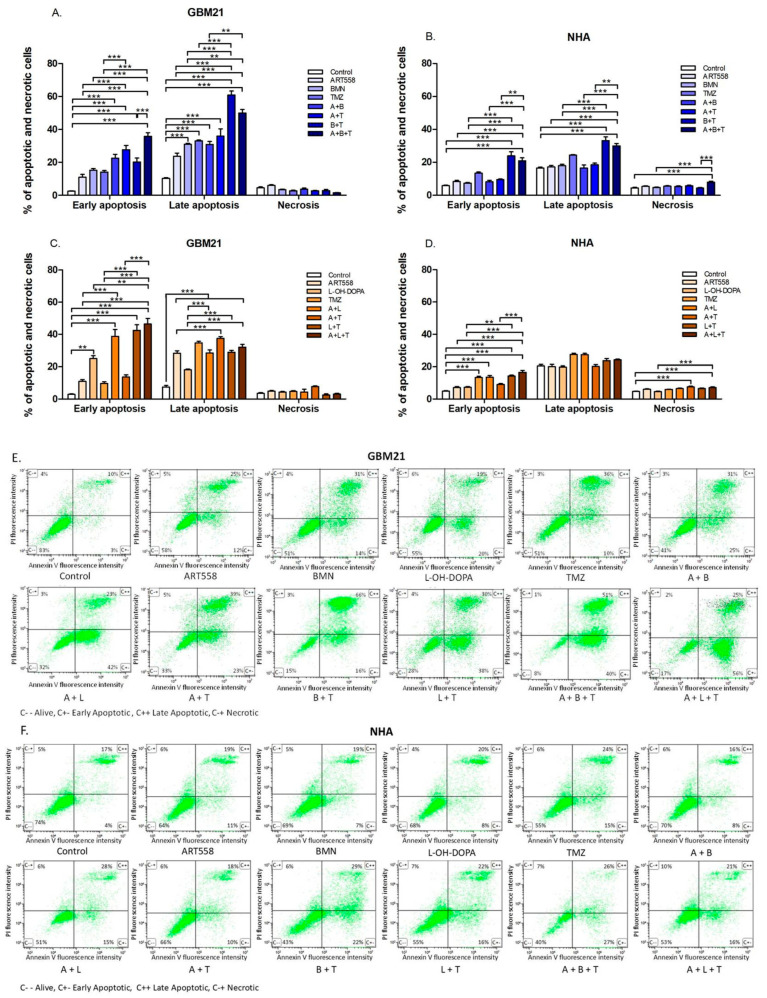
Proapoptotic effect of DNA repair protein inhibition and an alkylating agent and their combinations on the GBM21 cancer cell line. (**A**)—variant with PARPi, (**C**)—variant with RAD52i and normal NHA (**B**)—variant with PARPi, (**D**)—variant with RAD52i cells, shown as a percentage of cells in the corresponding stage in graphs and representative dot-plots (**E**)—GBM21, (**F**)—NHA. Cells were stained with propidium iodide (PI) and annexin V. Annexin V has strong affinity to phosphatidylserine, which appears on the cell’s surface during early apoptosis, while propidium iodide binds to DNA by penetrating through the fragmented cell membrane, which is characteristic of necrosis and the late stages of apoptosis. Three independent experiments were performed, and the results are shown as the mean ± standard error of the mean (SEM). ** *p* ≤ 0.01, *** *p*-value ≤ 0.001; A—ART558, B—BMN673, L—L-OH-DOPA, T—TMZ. C−−—Living cells, C+−—Early Apoptotic cells, C++—Late Apoptotic cells, C−+—Necrotic cells.

Double staining with PI and FITC Annexin V was used to determine whether the compounds induced apoptosis. Most of the cancer cells after treatment are in the stage of late apoptosis, some of them are in early apoptosis, and a small number are in necrosis, which indicates that inhibitors and TMZ lead to apoptotic cell death. The percentage of cancer cells in early apoptosis was significantly increased after the treatment with a combination of Polθi and PARPi or RAD52i, Polθi and TMZ, and the three compounds together in comparison to the control and separate inhibition of Polθ. A similar correlation was observed in the cell population in the late stage of apoptosis, but only in comparison to the control. In GBM21 differences between variants of treatment and control were not detectable in the case of necrosis (Figure 3A,C). Interestingly, a higher population of necrotic cells were observed in NHA than GBM21, suggesting that normal cells are less likely to undergo apoptosis. Moreover, dual inhibition of Polθ and PARP with TMZ significantly increased the necrotic population of normal cells. The combined treatment with Polθi and PARPi and TMZ significantly elevated the level of NHA cells in early and late apoptosis in comparison to the control, and separate inhibition of Polθ and Polθ with PARP in the case of late apoptosis (Figure 3B). The combined treatment with Polθi and RAD52i with or without TMZ gave a significant increase in NHA cells only in early apoptosis, compared to control and Polθ inhibition alone (Figure 3D). Described results are also illustrated with dot-plots (Figure 3E,F).

### 2.2. Inhibition of Polθ and Coinhibiton with Either PARP1 or Rad52 Decreases Proliferation of the Glioblastoma Cells

**Figure 4:**
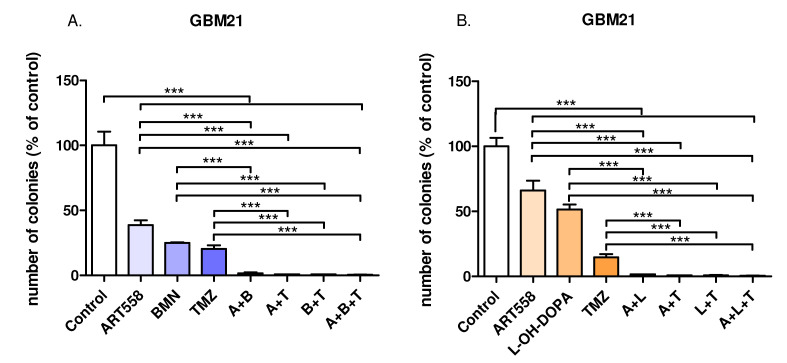

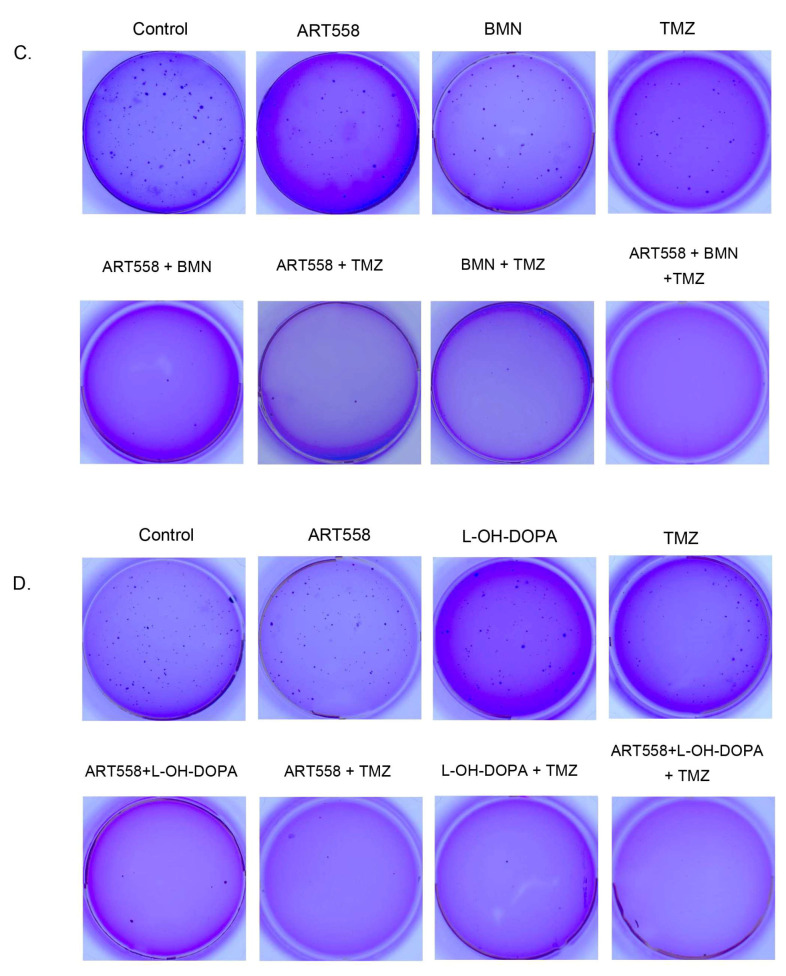
Reduction in colony formation induced by DNA repair protein inhibition and an alkylating agent and their combinations in GBM21 cells shown in graphs (**A**)—variant with PARPi, (**B**)—variant with RAD52i and photos of the representative plate wells (**C**)—variant with PARPi, (**D**)—variant with RAD52i. Three independent experiments were performed, and the results are shown as the mean ± standard error of the mean (SEM). *** *p*-value ≤ 0.001; A—ART558, B—BMN673, L—L-OH-DOPA, T—TMZ.

We used the clonogenic assay to measure the ability of single cancer cells to proliferate and form colonies after treatment with the inhibitors and the cytotoxic drug. Decreased cell proliferation, visualized as a significant reduction in colony formation, was observed between the control and each treatment variant. Also, in comparison to the single inhibition of Polθ, the combination of Polθi and PARPi or RAD52i or dual inhibition with TMZ gave statistically significant reduction in colony number. The results are also presented in the photos (Figure 4C,D).

### 2.3. Combined Inhibition of Polθ and Either PARP1 or Rad52 and Induction of DNA Alkylation by TMZ Leads to a Decrease in G0/G1 and Arrest of Cell Populations in S Phase, in Both Cancer and Normal Cells

**Figure 5:**
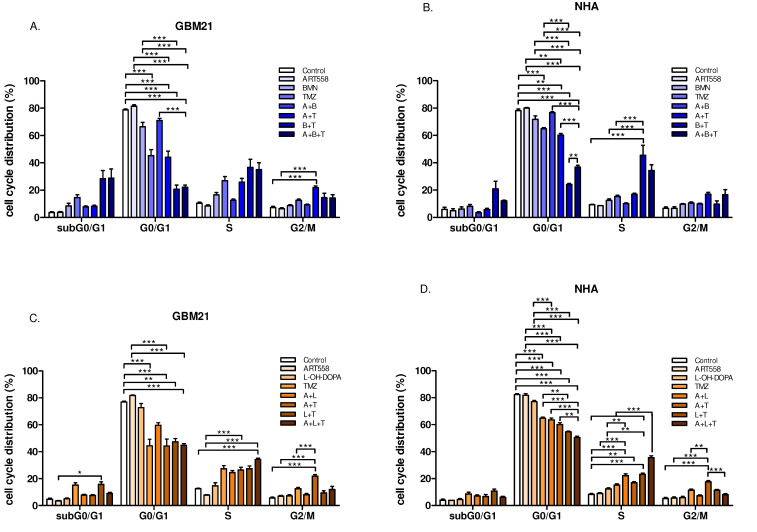
Distribution of cells in cycle phases after the inhibition of DNA repair proteins and alkylating agent treatment and their combinations in GBM21 (**A**)—variant with PARPi, (**C**)—variant with RAD52i and NHA (**B**)—variant with PARPi, (**D**)—variant with RAD52i cells. At least two independent experiments were performed, and the results are shown as the mean ± standard error of the mean (SEM). ** *p* ≤ 0.01, *** *p*-value ≤ 0.001; A—ART558, B—BMN673, L—L-OH-DOPA, T—TMZ.

In GBM21, the combination of Polθi with TMZ caused significant arrest of the cell cycle in G2/M and S phases and a decrease in G0/G1 in comparison to the control and Polθi (Figure 5A,C). Also, a significant effect on cell cycle changes was observed after treatment with RAD52i or PARPi and TMZ, i.e., a decrease in the G0/G1 cell population in comparison to the control in both cases and in single inhibition of PARP, in the case of PARPi with TMZ. The addition of Polθi to these treatments gave similar results, significant versus control and PARPi. Additionally, the combination of three compounds was significant compared to single inhibition of Polθ in the variant with RAD52i and to dual inhibition of PARP with Polθ in the variant of PARPi (Figure 5A,C). Further, the inhibition of Polθ and RAD52 with TMZ induced a statistically significant shift of the cells to S phase in comparison to the control and Polθi (Figure 5C). Similar results as shown for GBM21 were obtained for NHA cells (Figure 5B,D).

### 2.4. Coinhibition of Polθ with PARP1 Increases the Number of DSBs in the Glioblastoma Cells

**Figure 6:**
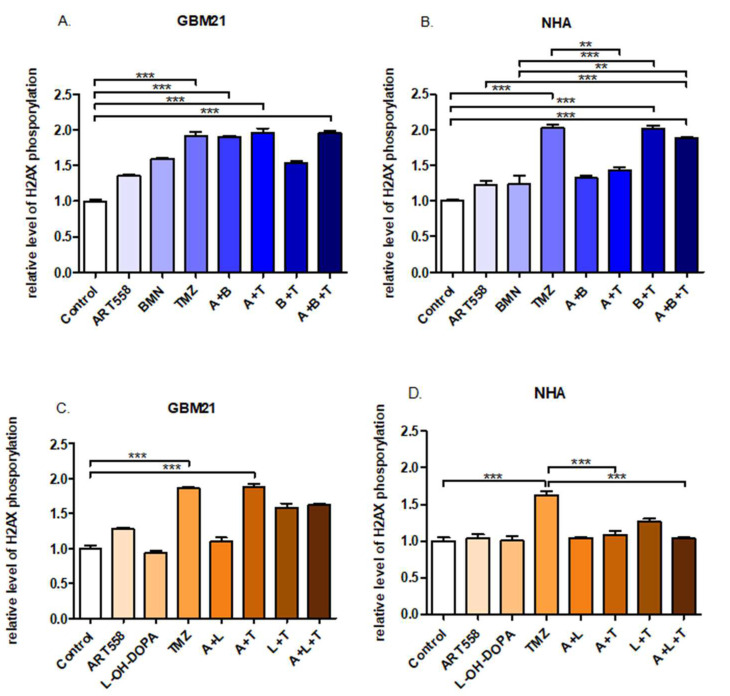
H2AX phosphorylation expressed as level of fluorescence signal relative to the control induced by the inhibition of DNA repair proteins and alkylating agent treatment and their combinations in GBM21 (**A**)—variant with PARPi, (**C**)—variant with RAD52i and NHA (**B**)—variant with PARPi, (**D**)—variant with RAD52i cells. At least two independent experiments were performed, and the results are shown as the mean ± standard error of the mean (SEM). ** *p* ≤ 0.01, *** *p*-value ≤ 0.001; A—ART558, B—BMN673, L—L-OH-DOPA, T—TMZ.

H2AX phosphorylation measurement served as a marker of DSBs. The results of this assay showed significant induction of H2AX phosphorylation in GBM21 after the dual inhibition of Polθ and PARP1 treatment and dual inhibition with TMZ in comparison to the control (Figure 6A). Also, separate usage of TMZ, as well as with Polθi induced DSBs at very similar levels, significant versus the control (Figure 6A,C). The NHA exhibited a significantly higher level of H2AX phosphorylation after the treatment with TMZ and the combination of Polθi and PARP1i and TMZ in comparison to the control and separate inhibition of Polθ.

### 2.5. Coinhibition of Polθ with PARP1 Enhances the Genotoxic Effect Obtained by Gamma Radiation in the Glioblastoma Cells, in Contrast to Normal Cells

**Figure 7:**
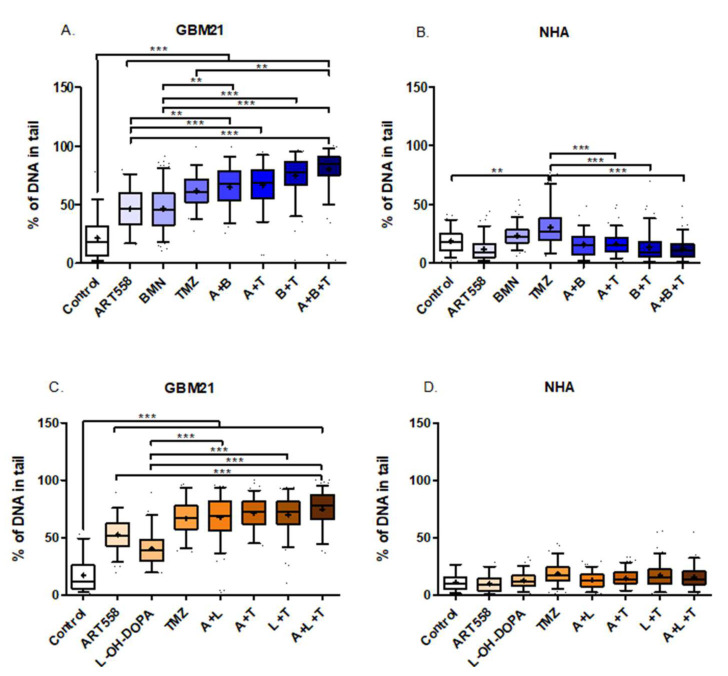
Double strand breaks induced by the inhibition of DNA repair proteins and alkylating agent treatment and their combinations in GBM21 (**A**)—variant with PARPi, (**C**)—variant with RAD52i and NHA (**B**)—variant with PARPi, (**D**)—variant with RAD52i cells, visualized as percentage of DNA in comet tails. Fifty randomly selected cells in three independent experiments were analyzed and are shown in box plots with whiskers representing 5–95th percentile. “+” shows mean value; ** *p* ≤ 0.01, *** *p*-value ≤ 0.001; A. In comparison to the control, all results are statistically significant with *p*-value ≤ 0.001; A—ART558, B—BMN673, L—L-OH-DOPA, T—TMZ.

The amount of DNA in comet tail is a direct marker of DNA damage, specifically double strand breaks, when the neutral version of the assay is considered. The exposure of the cells to gamma radiation was applied to simulate radiotherapy and analyze its combination with the used compounds. The percentage of DNA in the tail in GBM21 cells was significantly elevated between all variants of the treatment and control, shown in Figure 7A,C. Also, these cells exhibited significantly higher levels of DNA in the tails after the dual inhibition of Polθ and PARP1, and dual inhibition with TMZ in comparison to separate treatment with Polθi. Similarly, the combination of Polθ and RAD52 inhibition with TMZ gave a significant increase in DNA damage in comparison to the separate treatment with these inhibitors. Observing a relatively high level of DSBs after the combination of the treatment with radiation, we assume that Polθ inhibition in all variants can sensitize cells to radiotherapy. The percentage of DNA in the tail after the treatment in NHA cells was much lower than in cancer cells, showing the low genotoxic effect of the treatment and the ability of the cells to repair DSBs after radiation. Figure 7B shows a significant increase in DNA damage only after the treatment with TMZ and the results in Figure 7D did not show statistically significant differences.

## 3. Discussion

The inhibition of Polθ in the context of cancer treatment has been broadly investigated for the last few years [25,26,32,33,34,35,36,37,38]. In this study we determine that Polθ inhibition causes a significant viability decrease in glioblastoma cells, driven mainly by apoptosis, which was accompanied by a reduction in cell proliferation and DNA damage. Importantly, especially in the context of future application, the combined inhibition of Polθ with PARP1 or RAD52 boosts this anticancer effect in most of the presented experiments. Similarly, the simultaneous inhibition of Polθ with PARP1 or RAD52 was performed in a study on HR-deficient leukemias and showed a strengthened antileukemia effect, in comparison to the separate use of Polθi. The authors suggest that targeting two proteins with distinct repair activities, such as Polθ with PARP1 or RAD52, fosters synthetic lethality, which is consistent with our findings [36].

Further enhancement of this synergistic effect was observed in our study by supplementation with TMZ. Also, addition of TMZ to Polθi alone produces a significant decrease in glioblastoma viability and proliferation, and an increase in apoptosis and DNA damage in comparison to the separate Polθ inhibition and control group. Moreover, the combination of TMZ with Polθi shows a protective effect for NHA cells, observed by increased cell viability, a decreased population of cells in late apoptosis and lower level of DSBs than after separate use of TMZ. Together, these results point to the next direction for the future development of such treatments. 

Importantly, the results show that normal human astrocytes are impacted by the compounds to a much lower degree, but they are still influenced, especially by TMZ and the combination of the three compounds (PARPi or RAD52i variant). Such treatments significantly decreased cell viability and increased the rate of apoptosis and the H2AX phosphorylation level. The deleterious activity of TMZ is expected due to its alkylating mechanism, which is not specific to cancer cells, but to all cells in an organism [7,8,9]. Interestingly, the cell cycle distribution profile of NHA after all variants of the treatment is similar to that of glioblastoma cells.

Also, it is important to highlight that GBM21 cells have a relatively high expression of POLQ when compared to normal cells (Supplementary Figure S1), while a downregulation of any gene encoding DSB repair proteins was not determined. This could be one of the reasons why combined inhibition of Polθ with PARP1 or RAD52 kills cancer cells more effectively than inhibition of Polθ separately, while the effect of the treatment is less profound in normal cells. Concordantly, other research groups demonstrated that HR-deficient cells are more sensitive to Polθ depletion [25,36,37,38]. Moreover, there are multiple reports that POLQ is upregulated in cancer cells, also those with HR-deficiency, which could correlate with higher sensitivity to Polθ inhibition [25,39,40]. 

Moreover, in the context of future possible therapies, the combination of applied treatment with radiation was examined, showing a synergistic genotoxic effect on cancer cells, with a much lower impact on normal cells and almost no difference between variants of the treatment. The research of Rodriguez–Berriguete et al. (2023) indicates supporting results that ART558, by inhibition of Polθ, sensitizes cancer cells to radiotherapy, independently of HR-deficiency [41]. In addition, other studies show that human and mouse cancer cells with Polθ depletion are more sensitive to gamma radiation [42,43]. These findings could be correlated with the involvement of Polθ in genome stability maintenance [44,45,46]. Thus, it can be another potential approach for developing a therapy based on Polθ inhibition.

The research of Ronson and Starowicz [37,38], identified an interesting interaction during Polθ inhibition in 53BP1-, USP48- deficient cells and Polθ depletion in 53BP1- and BRCA1/2-deficient cells, where RAD52 enhances its toxicity by promoting DNA resection, chromosome breaks, DSBs and cell death. The research suggests that moderate inhibition of RAD52 can help cells to survive, while high-concentration inhibition of RAD52 during Polθ inhibition/depletion would be too harmful, confirming a synthetic lethality interaction between Polθ and RAD52 [37,38]. The interaction triggered by simultaneous inhibition of Polθ and RAD52 also appeared in our studies. However, due to an unknown 53BP1 gene expression, it was not possible to assess if a similar mechanism is the cause of this effect.

Moreover, the drug resistance, which is an already known and studied drawback of PARP inhibitors, is also an important issue to address. The hope to overcome this problem is placed in Polθ inhibition [22,25,26,47,48]. Zatreanu et al. (2021) and Zhou et al. (2021) were the first to introduce ART558 and Novobiocin, respectively, as Polθ inhibitors with very promising results, and also in PARPi resistant tumors [25,26]. Zatreanu et al. (2021) indicated that Polθ inhibition could be used to overcome PARPi resistance acquired by depletion of Shieldin complex components in BRCA1−/− cells by a mechanism of dual synthetic lethality, which also agrees with the results obtained by Zhou et al. (2021) [25,26]. Apart from the mutation/depletion of the Shieldin complex mentioned above, PARPi resistance can be caused by: (i) reversion mutations in BRCA1/2 genes, leading to restoration of functional protein and the HR pathway; (ii) involvement of mutations in the 53BP1 protein; (iii) mutation in PARP itself; and (iv) pharmacokinetic changes in the drug. These scenarios are also possible in the context of Polθ inhibition, however, it is difficult to predict them. The solution for this issue, similar to PARPi resistance, could be the combination of Polθ inhibition with inhibition of other DNA repair proteins [22]. The results of this research show that the combination of Polθ inhibition with either PARPi or RAD52i intensifies the cytotoxic and genotoxic effect in glioblastoma, to a lower extent than in astrocytes. Thus, it may be a way to evade cells’ resistance, however further research on a resistant cell line would be recommended.

Summarizing, the synthetic lethality achieved by simultaneous inhibition of Polθ and PARP or Polθ and RAD52 is a promising approach to eliminate glioblastoma cells. Nevertheless, many challenges must be overcome, such as dosing, toxicity to healthy tissues and cell resistance, before it could be implemented as a patient treatment.

## 4. Materials and Methods

### 4.1. In Vitro Cell Culture

A glioblastoma cell line derived from a surgical specimen, was obtained from a patient of the Department of Neurosurgery, Surgery of Spine and Peripheral Nerves, University Hospital WAM-CSW Lodz. The cell line was established in the Laboratory of the Medical Biochemistry Department, Medical University of Lodz and named GBM21. The study was approved by the Ethical Commission of the Medical University of Lodz (RNN/23/22/KE) and informed consent was obtained from all patients. To obtain the cell line, tissue fragments were washed several times with HBSS buffer (Gibco, Thermo Fisher Scientific, Waltham, MA, USA) and minced mechanically with a scalpel under sterile conditions. The shredded tissue fragments were passed through a filter with a pore size of 70 μm, and then centrifuged. If a large number of red blood cells were present in the cell pellet, RBC lysis buffer (Sigma-Aldrich, Saint Louis, MO, USA) was used.

Glioblastoma cells were cultured in DMEM/F12 medium (Gibco, Thermo Fisher Scientific, Waltham, MA, USA) supplemented with 10% FBS (Lonza, Basel, Switzerland), 100 IU/mL penicillin, 100 µg/mL streptomycin (Lonza, Basel, Switzerland) and gentamycin, 50 µg/mL (Lonza, Basel, Switzerland) in a humidified atmosphere containing 5% CO2 at 37 °C. Once the cells had multiplied, they were subjected to positive selection via magnetic-activated cell sorting on a magnetic separator (MiniMACS™, Miltenyi Biotec, Bergisch Gladbach, Germany) with use of CD133 magnetic microbeads MACS^®^ (Miltenyi Biotec, Bergisch Gladbach, Germany). The Normal Human Astrocytes—NHA (Lonza, Basel, Switzerland)—were grown in ABM^TM^ Basal Medium supplemented with AGM^TM^ SingleQuots^TM^ Supplements (Lonza, Basel, Switzerland) and cultured according to the protocol provided by the manufacturer.

The GBM21 cell line was tested for *POLQ* gene expression and exhibited overexpression compared to NHA cells.

### 4.2. Drug Treatment

In the experiments, the following compounds were used: inhibitor of Polθ—ART558 (MedChem Express, Monmouth Junction, NJ, USA), 56.5 μM; inhibitor of PARP1—talazoparib (BMN673) (Selleckchem, Houston, TX, USA), 110 nM; inhibitor of RAD52—L-OH-DOPA (Sigma-Aldrich), 62.5 μM; and alkylating drug—temozolomide (TMZ), 37 μM. We established doses of the compounds performing dose screening by measuring cell viability by flow cytometry prior to the main experiments. We aimed to maintain the concentration of each compound below the IC50 threshold. Compounds were dissolved appropriately according to the manufacturer’s instructions in distilled water or DMSO to a starting concentration of 10 mM, and then working concentrations were prepared immediately before the experiment in DMEM/F12 culture medium. The treatment scheme was established and proved to work in previous experiments. Briefly, cells were incubated with the compounds for 120 h with the second dose after 48 h [30,31,49,50]. Following variants of the treatment were used: ART558, BMN673, L-OH-DOPA and TMZ separately, ART558 + BMN673, ART558 + L-OH-DOPA, ART558 + TMZ, BMN673 + TMZ, L-OH-DOPA + TMZ, ART558 + BMN673 + TMZ, ART558 + L-OH-DOPA + TMZ.

### 4.3. Flow Cytometric Analysis of Apoptosis and Necrosis

Changes in viability and mechanism of cell death after standard treatment described above were analyzed using staining with propidium iodide and FITC Annexin V. Cells were prepared and analyzed according to the FITC Annexin Apoptosis Detection Kit II (BD Biosciences, Franklin Lakes, NJ, USA) by flow cytometry. Annexin V has strong affinity to phosphatidylserine, which appears on the cell’s surface during early apoptosis, while propidium iodide binds to DNA by penetrating through the fragmented cell membrane, which is characteristic of necrosis and the late stages of apoptosis. Cell viability results were also obtained using this assay.

### 4.4. Cell Morphology Visualized by Fluorescence Microscopy 

To visualize the influence of inhibitors on cell viability, normal and cancer cells were subjected to calcein AM and propidium iodide (PI) double staining. Cells were stained after standard treatment and incubated for 30 min at 37 °C with the mixture of 2 mM calcein AM and 1 mM propidium iodide (Thermo Fisher Scientific, Waltham, MA, USA) diluted in PBS. Calcein AM, the acetoxymethyl ester of calcein, freely penetrates the membranes of living cells, where the acetoxymethyl group is degraded, allowing calcium binding to calcein and showing strong green fluorescence, when excited. Propidium iodide stains the DNA of dead cells with low plasma membrane integrity, indicated by a red fluorescence signal. The results were observed and pictured on an inverted fluorescence microscope from LEICA.

### 4.5. Clonogenic Assay

The cancer cells’ ability to form colonies was measured by a clonogenic assay. Prior to the test, cells had undergone the standard treatment described above. Then, trypan blue staining was used to assess the viability of the treated cells. Following this, 10^3^ cells were resuspended in 700 µL of 0.4% soft agar containing DMEM, FBS and antibiotics and plated on a 12-well plate over 700 µL of solidified agar underlay (0.5%, also with DMEM, FBS and antibiotics). The medium was applied over the solidified cell layer and changed weekly. Prepared plates were incubated for 2 weeks, at 37 °C, 5% CO_2_. The colonies were stained with 0.005% crystal violet and counted under the microscope. Clonogenic efficiency was expressed as percent of untreated control (no. of colonies after treatment vs no. of colonies in control sample × 100%).

### 4.6. Cell Cycle

To analyze the influence of the compounds on cell cycle distribution in glioblastoma and NHA populations, cells fixed with 70% cold ethanol were stained with propidium iodide with the addition of RNase (BD Biosciences) and analyzed by flow cytometry.

### 4.7. Measurement of Histone H2AX Phosphorylation

The levels of phosphorylated histone H2AX, constituting DNA DSBs, were measured by the H2AX Phosphorylation Assay Kit (Merck KGaA, Darmstadt, Germany). After the standard treatment, the cells were fixed and permeabilized to facilitate staining and detection. The presence of histone H2AX phosphorylated at serine 139 was detected using a FITC-conjugated anti-phospho-histone H2AX antibody. Flow cytometry was employed to quantify the number of cells exhibiting positive staining for phosphorylated histone H2AX.

### 4.8. Neutral Comet Assay

The level of DNA double-strand breaks generated by the used compounds was studied using the neutral comet assay. Following the standard treatment, cells were exposed to gamma radiation of 8 Gy. After that, cells were resuspended in 0.4% low melting point (LMP) agarose solution and immediately applied to the precoated slide with 0.5% normal melting point (NMP) agarose. Prepared slides were subjected to overnight lysis (2.5 M NaCl, 100 mM EDTA, 10 mM TRIS). Then, the slides were placed in developing buffer (300 mM/L NaOH, 1 mM/L EDTA) for 20 min. After that, electrophoresis was carried out in the electrophoretic buffer (300 mM sodium acetate, 100 mM TRIS) for 1 h, at 9 V and 100 mA. After finishing electrophoresis, the slides were rinsed with water and stained with DAPI solution (100 μg/mL) by applying 50 μL of solution to each slide and incubating the slides for at least 45 min, at 4 °C. To visualize the results, the slides were observed at 200× magnification on an Eclipse fluorescence microscope (Nikon, Tokyo, Japan) attached to a COHU 4910 video camera (Cohu, Inc., San Diego, CA, USA) equipped with a UV-1 A filter block and connected to a personal computer-based image analysis system, Lucia-Comet v. 6.0 (Laboratory Imaging, Praha, Czech Republic). Fifty comets were counted from each repetition of the experiment. The % of the DNA in the comet tail was taken into account. 

### 4.9. Statistical Analysis

Data from at least three independent experiments were analyzed and presented as mean ± SEM. The results were compared in SigmaPlot, using one-way ANOVA with the Holm–Sidak post hoc test. All graphs were created in GraphPad Prism 10. *p*-values of <0.05 were considered statistically significant.

## 5. Conclusions

Presented and discussed results show that targeting Polθ in cancer treatment has a promising potential and should be further explored. The extensive work on research and development of new and more efficient Polθ inhibitors continues, along with the use of AI, which could enhance this process, but should be discussed in a broader dedicated manuscript [34,48]. The synthetic lethality achieved by combined inhibition of Polθ with other DNA repair proteins, such as PARP1 or RAD52, have an even greater effect on defeating glioblastoma; however, it needs to be studied in the broader context of its toxicity. In our opinion, the addition of the alkylating agent temozolomide could significantly increase efficiency, but also toxicity, of the potential treatment with these inhibitors. 

To have the potential for application in real cancer treatments, the results should be expanded by in vitro studies in more cell lines and in in vivo experiments, followed by clinical studies on humans. It is worth noticing, as a limitation of the study, that it was performed on only one patient-derived cancer cell line, therefore it cannot be directly scaled up, however, it points out the role of personalized medicine. Also, more extensive analysis of glioblastoma cell genetic profiles is necessary to reveal the correlations which lie beneath the antitumor activity of Polθ inhibition.

## Data Availability

The data presented in the study are available upon request.

## Supplementary Materials

The following supporting information can be downloaded at: https://www.mdpi.com/article/10.3390/ijms25179134/s1.
