# Correction: Barszczewska-Pietraszek et al. Polθ Inhibitor (ART558) Demonstrates a Synthetic Lethal Effect with PARP and RAD52 Inhibitors in Glioblastoma Cells. *Int. J. Mol. Sci.* 2024, *25*, 9134

**DOI:** 10.3390/ijms27031327

**Published:** 2026-01-29

**Authors:** Gabriela Barszczewska-Pietraszek, Piotr Czarny, Małgorzata Drzewiecka, Maciej Błaszczyk, Maciej Radek, Ewelina Synowiec, Paulina Wigner-Jeziorska, Przemysław Sitarek, Janusz Szemraj, Tomasz Skorski, Tomasz Śliwiński

**Affiliations:** 1 Department of Molecular Genetics, Faculty of Biology and Environmental Protection, University of Lodz, 90-236 Lodz, Poland; 2 Department of Medical Biochemistry, Medical University of Lodz, 92-216 Lodz, Poland; 3 Department of Neurosurgery, Surgery of Spine and Peripheral Nerves, Medical University of Lodz, University Hospital WAM-CSW, 90-549 Lodz, Poland; 4 Department of Medical Biology, Medical University of Lodz, 92-151 Lodz, Poland; 5 Fels Cancer Institute for Personalized Medicine, Lewis Katz School of Medicine, Temple University, Philadelphia, PA 19140, USA

In the original publication [1], there was a mistake in Figure 3 as published. The same dot plot was used for Figure 3E A + B and 3E A + L, although it is valid only for Figure 3E A + B. This oversight occurred inadvertently due to the processing of a large dataset. The corrected Figure 3 appears below. The authors state that the scientific conclusions are unaffected. This correction was approved by the Academic Editor. The original publication has also been updated.

## Figures and Tables

**Figure 3:**
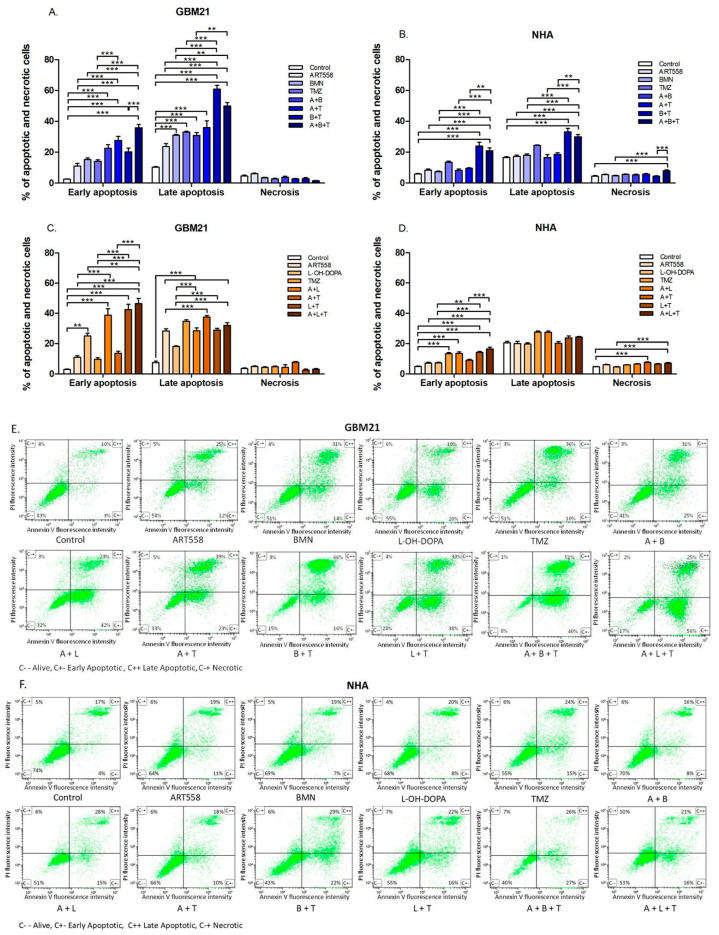
Proapoptotic effect of DNA repair protein inhibition and an alkylating agent and their combinations on the GBM21 cancer cell line. (**A**)—variant with PARPi, (**C**)—variant with RAD52i and normal NHA (**B**)—variant with PARPi, (**D**)—variant with RAD52i cells, shown as a percentage of cells in the corresponding stage in graphs and representative dot-plots (**E**)—GBM21, (**F**)—NHA. Cells were stained with propidium iodide (PI) and annexin V. Annexin V has strong affinity to phosphatidylserine, which appears on the cell’s surface during early apoptosis, while propidium iodide binds to DNA by penetrating through the fragmented cell membrane, which is characteristic of necrosis and the late stages of apoptosis. Three independent experiments were performed, and the results are shown as the mean ± standard error of the mean (SEM). ** *p* ≤ 0.01, *** *p*-value ≤ 0.001; A—ART558, B—BMN673, L—L-OH-DOPA, T—TMZ. C−−—Living cells, C+−—Early Apoptotic cells, C++—Late Apoptotic cells, C−+—Necrotic cells.
