# Supplementary material for: Polθ Inhibitor (ART558) Demonstrates a Synthetic Lethal Effect with PARP and RAD52 Inhibitors in Glioblastoma Cells
Source: Int J Mol Sci. 2024 Aug 23;25(17):9134. doi: 10.3390/ijms25179134 (PMC11395082; doi:10.3390/ijms25179134)
Supplement: Supplementary file 1 [file ijms-25-09134-s001.zip › ijms-3088553-supplementary.pdf]

**Glioblastoma GBM21 gene expression**

**Methodology : RNA isolation and gene expression**
Analysis of mRNA expression of 28 selected genes, which products are involved in following DSB repair mechanisms: HR, NHEJ, TMEJ and a-NHEJ, began with the isolation of total RNA using RNeasy Mini Kit (Qiagen, Venlo, The Netherlands), according to the manufacturer's instructions. After the detachment from culture bottles, cells were centrifuged and then resuspended in PBS to determine cell density. Approximately $5x10^6$ cells were used for isolation, with a survival rate of 97-99%.
Subsequently, the purity and quantity of RNA were evaluated by spectrophotometric analysis in Picodrop, then 10 ng/µL RNA was converted into complementary DNA (cDNA), using High Capacity cDNA Reverse Transcription Kit (Thermo Fisher Scientific, Waltham, Massachusetts, USA). Real-time PCR was performed with TaqMan® probes dedicated to detect 4 reference and the selected genes. The RT-PCR reactions were conducted on CFX96 ™ Real-Time PCR Detection System (Bio-Rad Laboratories, Hercules, California, USA) with parameters consisted of an initial step of 95 ◦C for 10 min, followed by 30 cycles of denaturation at 95 ◦C for 15 s and annealing/extension at 60 ◦C for 60 s., using TaqMan™ Universal Master Mix II probes, no UNG (Thermo Fisher Scientific, Waltham, Massachusetts, USA). Each reaction was performed in triplicates. *ACTB* was used as a reference gene after analysis in Geneinvestigator where it was established with the most stable expression between 4 selected reference genes. The results were calculated as fold change of genes expression in cancer versus normal cells ($2^{-\Delta\Delta Ct}$).

**Description of the reuslts***:* To assess genetic profile of cancer cell line and potential deficinency in any of repair pathway we determine expression level of 28 genes, selected due to their activity in DSBs repair pathways. We did not observed a downregulation of any recalled gene versus NHA cells. However, relatively high expression of HR-related genes and *POLQ* of TMEJ, while all genes involved in NHEJ have realtively low expression. Therefore, this may explain higher effectivness of dual inhibition with Polθi and PARPi or RAD52i.

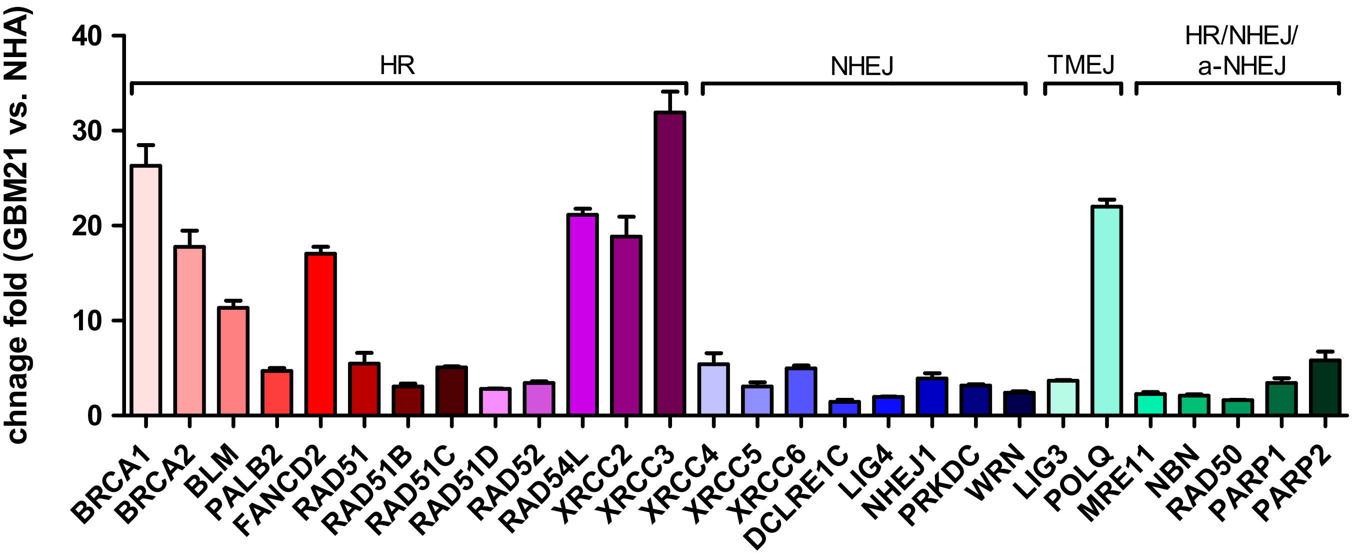


Figure S1 Expression profile of 28 genes, categorized due to their activity in following DNA double strand breaks repair mechanisms: homologous recombination (HR), non-homologous end joining (NHEJ), theta-mediated end joining (TMEJ), alternative non-homologous end joining (a-NHEJ) in GBM21 glioblastoma cells, presented as a fold change in reference to normal cells NHA. Results represent mean value ± SEM from the experiment performed in triplicate.
